# Supplementary material for: Metabolic Profile, Biotransformation, Docking Studies and Molecular Dynamics Simulations of Bioactive Compounds Secreted by CG3 Strain
Source: Antibiotics (Basel). 2022 May 13;11(5):657. doi: 10.3390/antibiotics11050657 (PMC9137728; doi:10.3390/antibiotics11050657)
Supplement: Supplementary file 1 [file antibiotics-11-00657-s001.zip › antibiotics-1701318-supplementary.pdf]

Supporting Information

Metabolic Profile, Biotransformation, Docking Studies and Molecular Dynamics Simulations of Bioactive Compounds Secreted by CG3 Strain

Omar Messaoudi [1,2,3], **Enge Sudarman** [4,5], **Chirag Patel** [6], **Mourad Bendahou** [3] and **Joachim Wink** [2,*]

1 Department of Biology, Faculty of Science, University of Amar Telidji, 03000 Laghouat, Algeria; o.messaoudi@lagh-univ.dz
2 Microbial Strain Collection, Helmholtz Centre for Infection Research GmbH (HZI), Inhoffenstrasse 7, 38124 Braunschweig, Germany
3 Laboratory of Applied Microbiology in Food and Environment, Abou Bekr Belkaïd University, 13000 Tlemcen, Algeria; bendahou63@yahoo.fr
4 Department Microbial Drugs, Helmholtz Centre for Infection Research GmbH (HZI), Inhoffenstrasse 7, 38124 Braunschweig, Germany; e.sudarman@web.de
5 German Centre for Infection Research Association (DZIF), Partner site Hannover-Braunschweig, Inhoffenstrasse 7, 38124 Braunschweig, Germany
6 Computer-Aided Drug Design Group, Chemical Biology Laboratory, Center for Cancer Research, National Cancer Institute, National Institute of Health, Frederick, MD 21702, USA; chiragpatel269@gmail.com
* Correspondence: joachim.wink@helmholtz-hzi.de

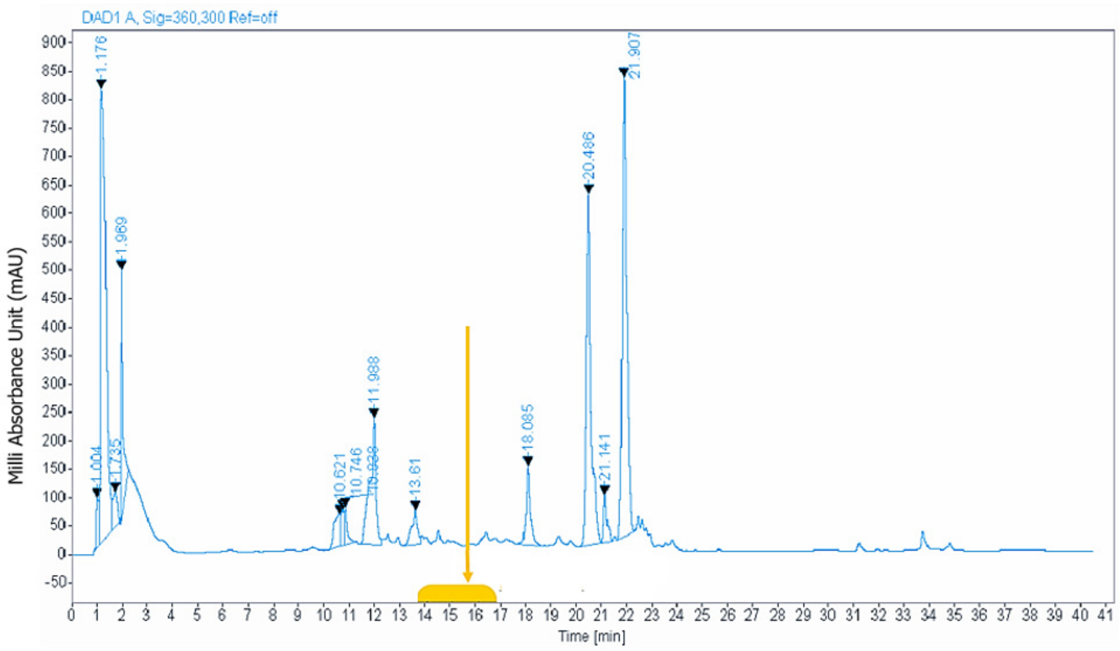

Figure S1. HPLC chromatogram of the crude extract prepared from the culture of strain CG3 in ISP2 medium, indicate the absence of the three peaks **50, 52** and **54** eluted between t_R :14.50-16.50 min.

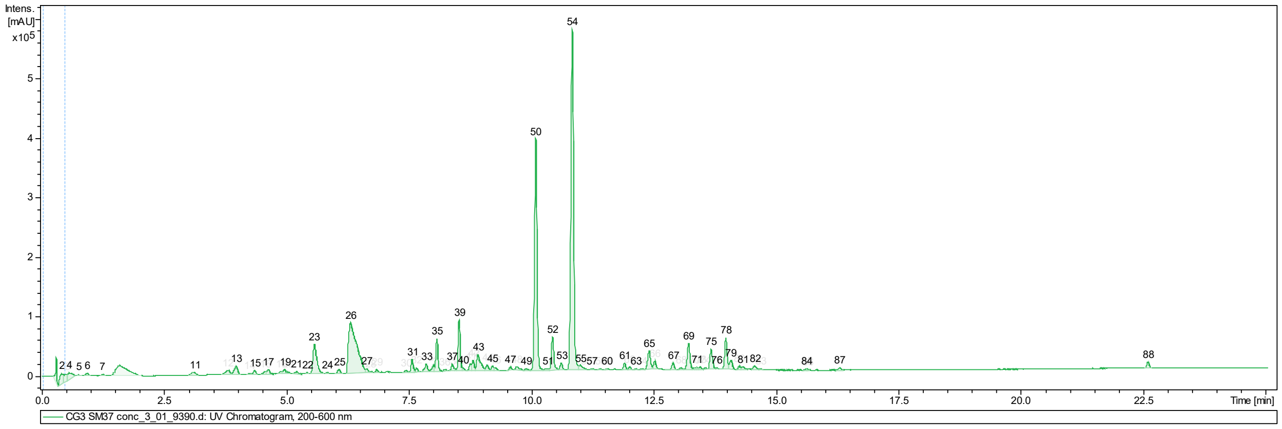

Figure S2. LC-UV-MS profile of crude extract prepared from the culture of strain CG3 in SM medium.

Compound 13

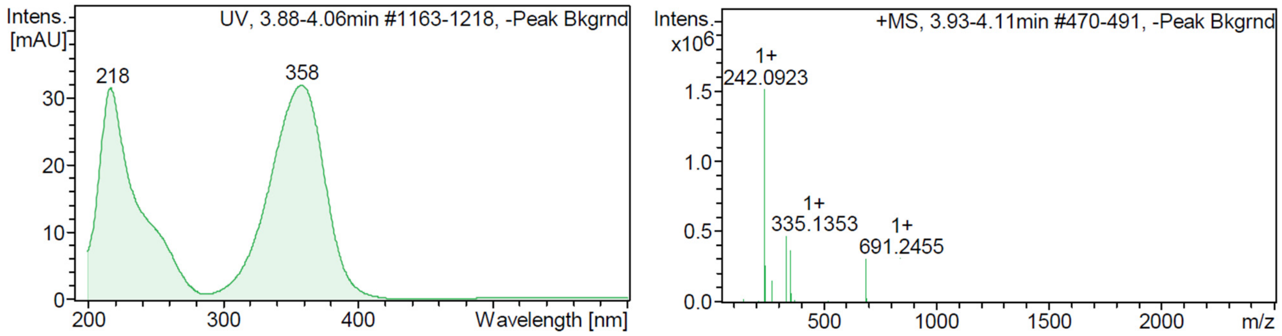

Compound 39

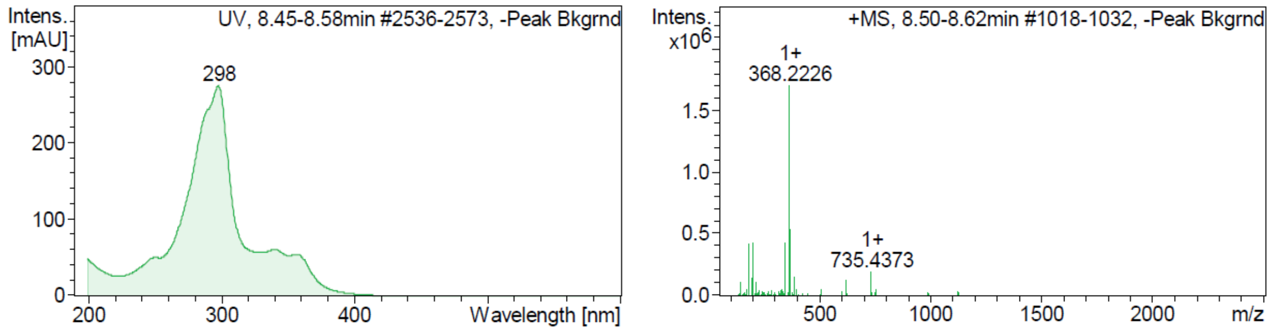

Compound 50

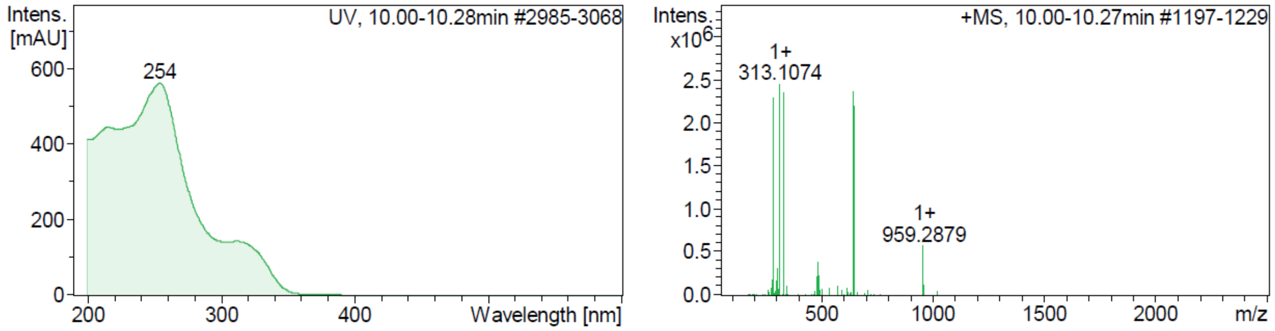

Compound 52

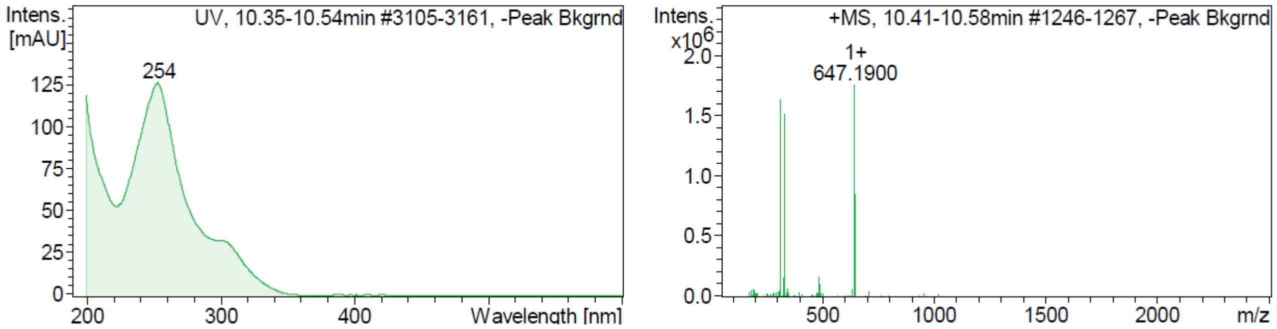

Compound 54

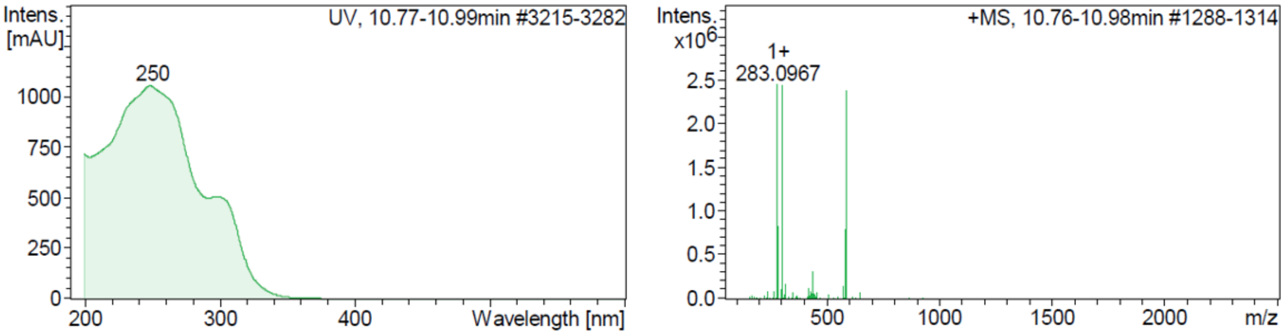

Compound 65

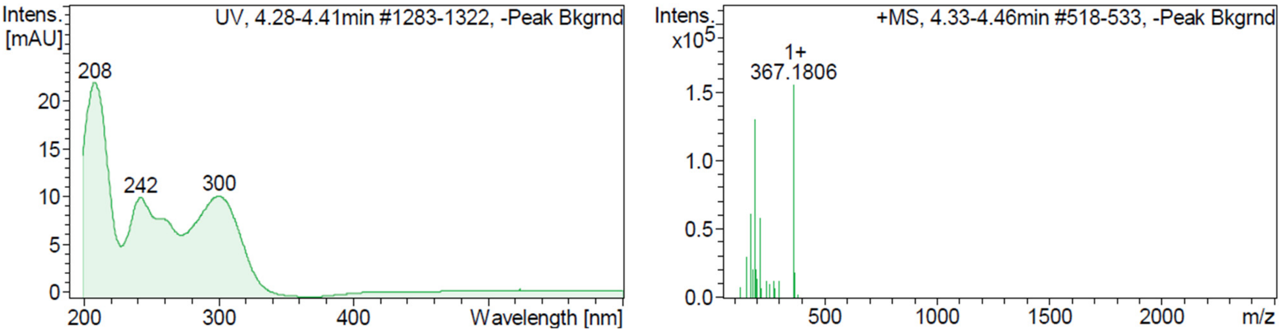

Compound 69

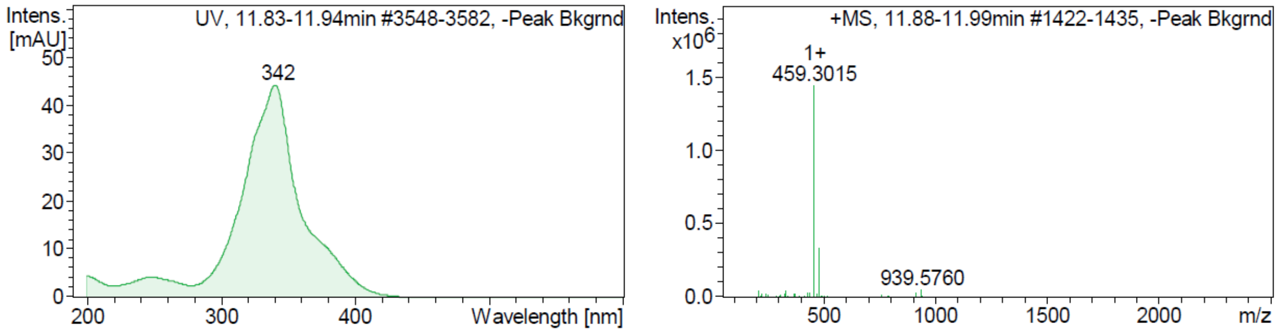

Compound 75

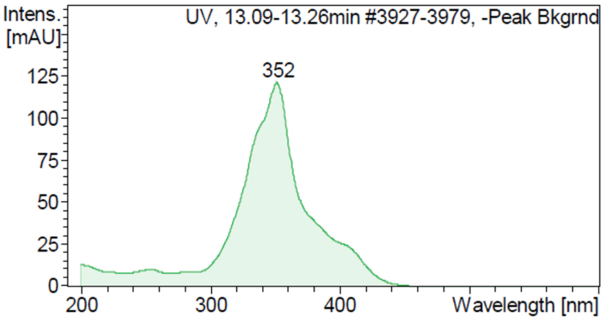

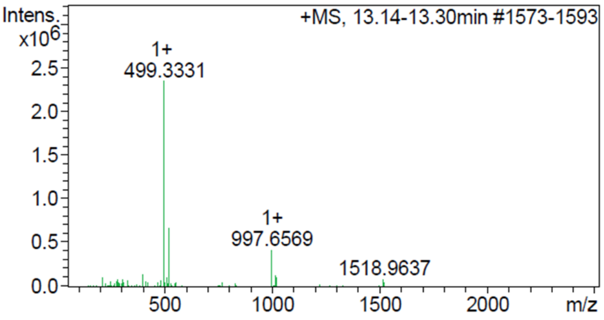

Compound 78

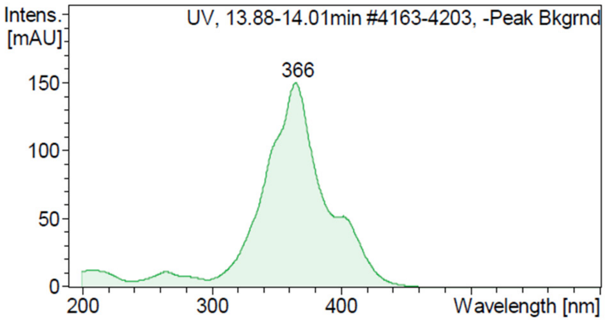

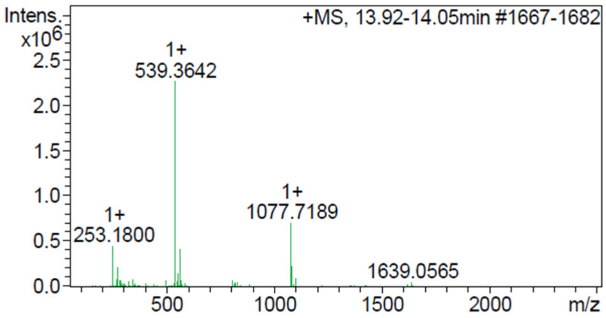

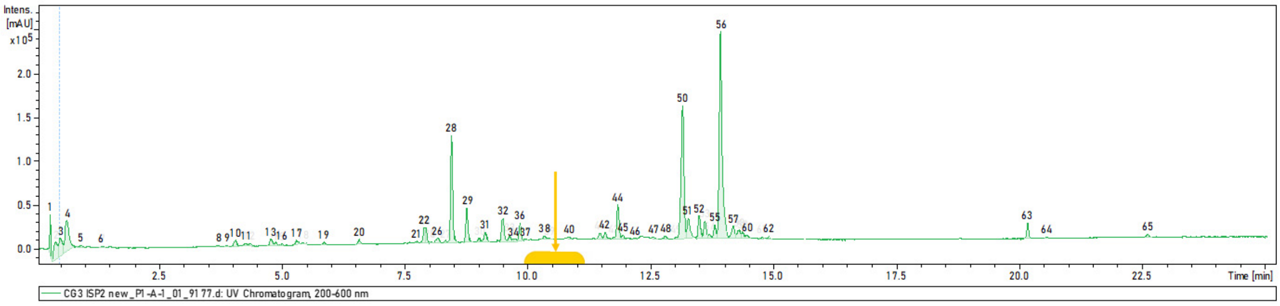

Figure S3. LC-UV-MS profile of the crude extract of strain CG3 cultured in ISP2 medium, indicat the absence of the three peaks **50**, **52** and **54**.

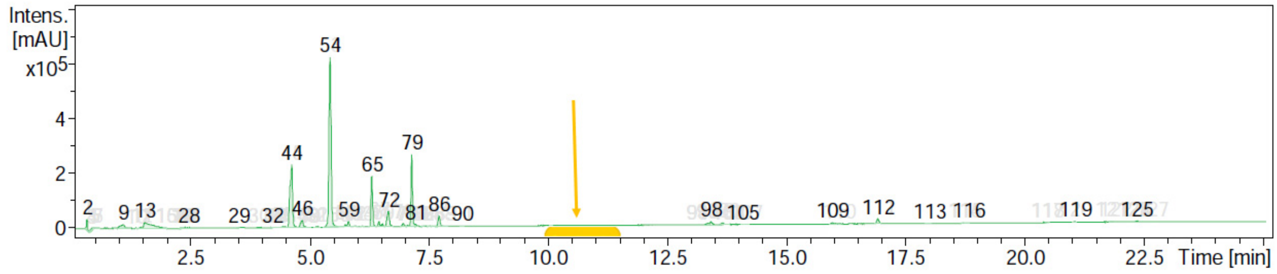

Figure S4. LC-UV-MS profile of crude extract prepared from SM without the strain CG3 indicat the absence of the three peaks **50**, **52** and **54**.

Compound 44: Genistein

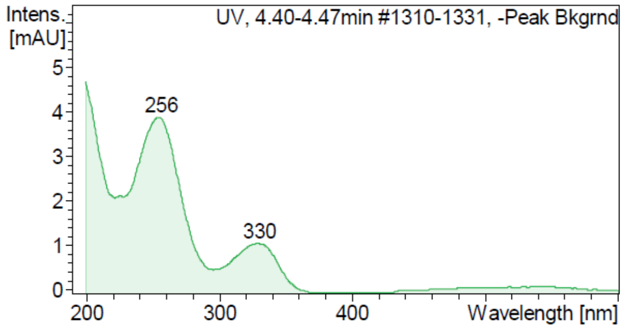

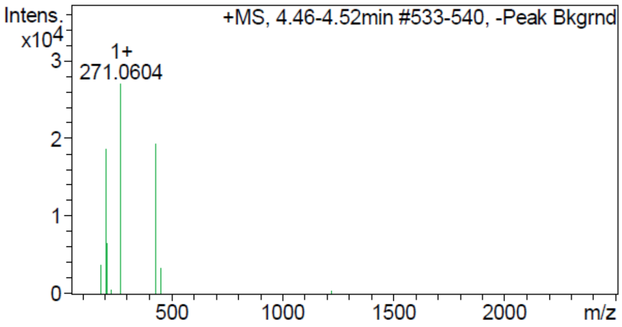

compound 46: Glycitein

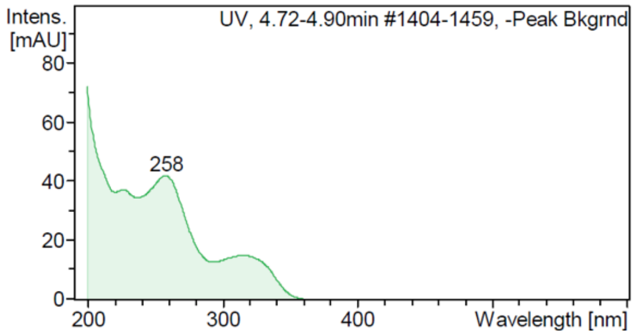

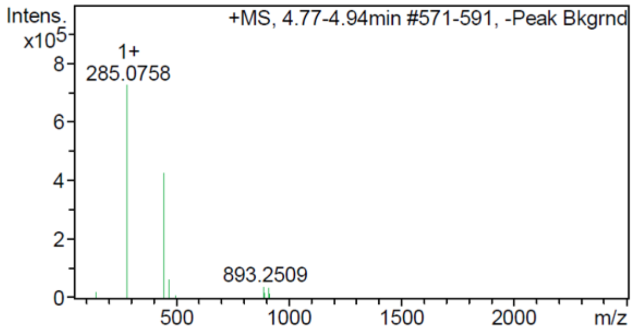

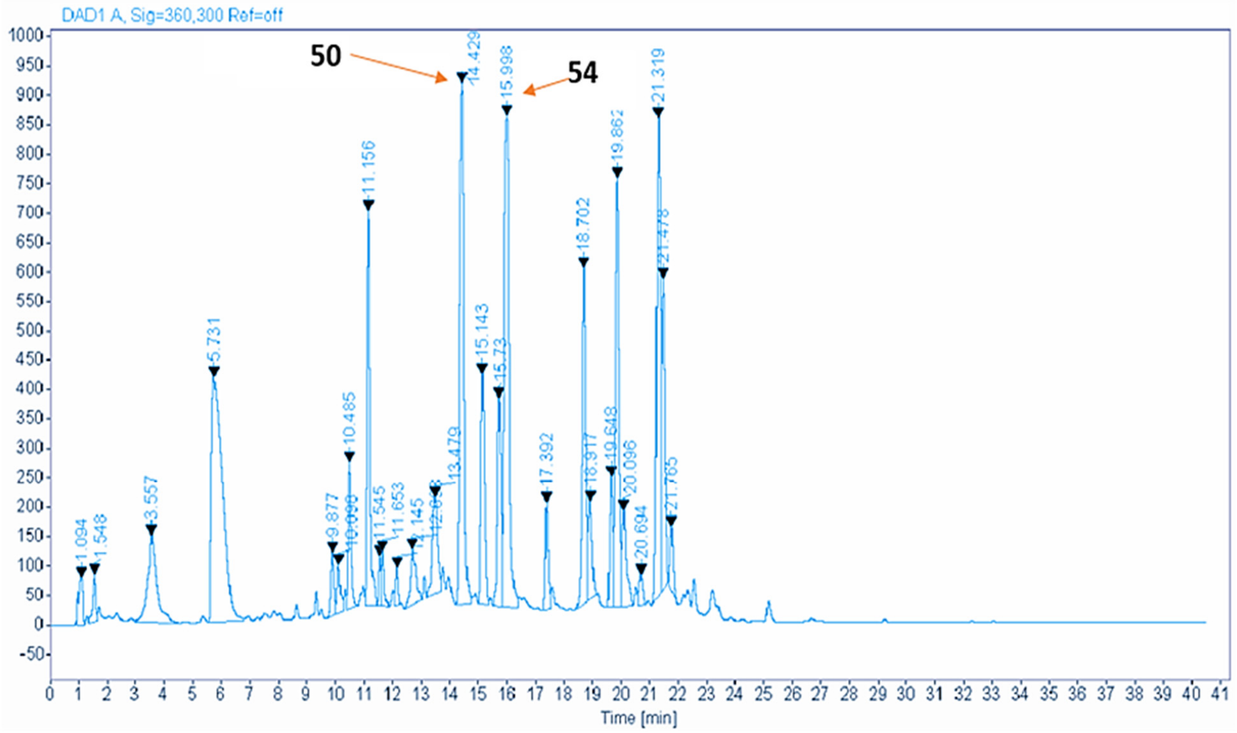

Figure S5. HPLC chromatogram of the crude extract prepared after 10 days incubation of strain CG3 cultured in SM.

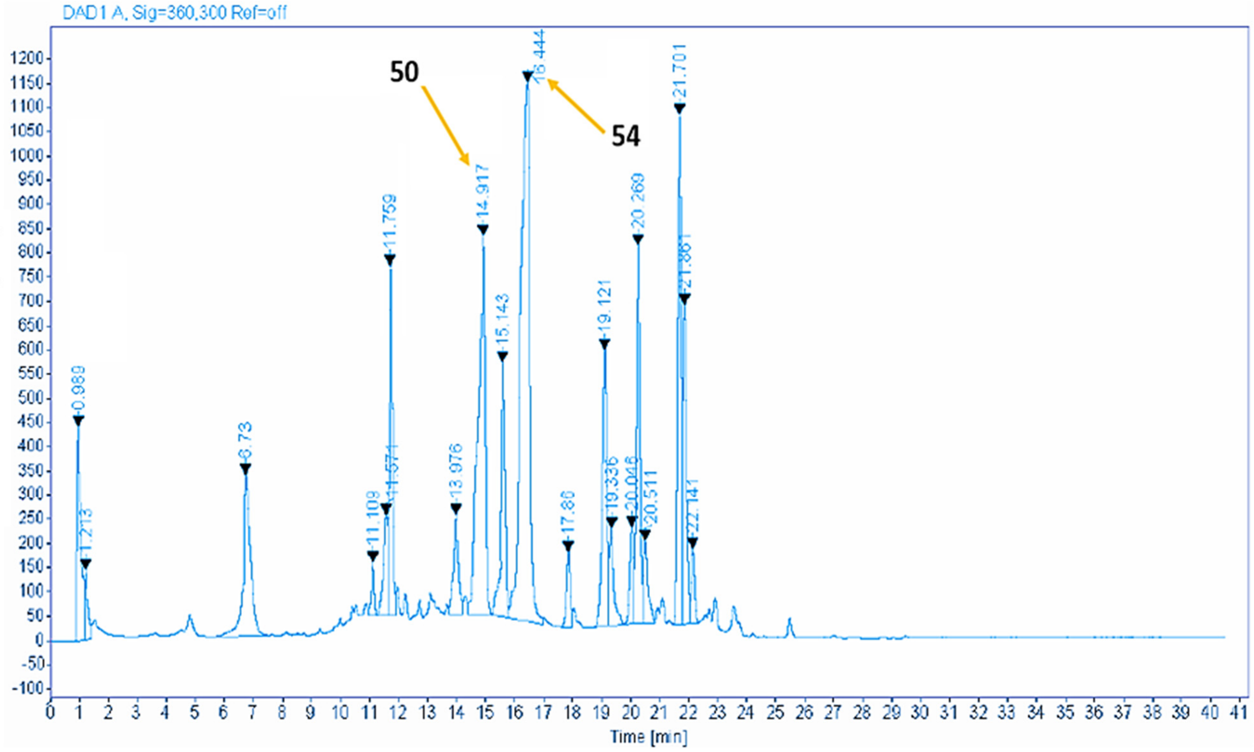

Figure S6. HPLC chromatogram of the crude extract prepared after 21 days incubation of strain CG3 cultured in SM.

NMR (^{1}H 700 MHz, ^{13}C) data of compounds: 65,7-dimethoxy-3-(4-methoxyphenyl)chromen-4-one (50); 6,7-dimethoxy-3-phenylchromen-4-one (54) and 5'-hydroxy-2',3',4,4''-tetramethoxy-p-terphenyl (65), in CD_3OD-*d*$_4$.

Table S1. NMR data of 5,7-dimethoxy-3-(4-methoxyphenyl)chromen-4-one **(50)** in CD_3OD-*d*$_4$ (^{1}H 700 MHz, ^{13}C 176 MHz)

pos.	δ_C, type	δ_H,mult (*J* in Hz)
2	154.9, CH	8.24, s
3	125.7, C	
4	177.9, C	
4a	118.7, C	
5	105.5, CH	7.60, s
6	149.7, C	
7	156.8, C	
8	101.2, CH	7.16, s
1'	125.7, C	
2', 6'	131.6, CH	7.51, d (8.8)
3', 5'	115.0, CH	7.01, d (8.8)
4'	161.3, C	7.02, d (8.8)
6-OCH$_3$	56.8, CH$_3$	3.96, s
7-OCH$_3$	57.2, CH$_3$	4.00, s
4'-OCH$_3$	55.9, CH$_3$	3.86, s

Table S2. NMR data of 6,7-dimethoxy-3-phenylchromen-4-one **(54)** in CD_3OD-*d*$_4$ (^{1}H 700 MHz, ^{13}C 176 MHz)

pos.	δ_C, type	δ_H,mult (*J* in Hz)
2	154.9, CH	8.24, s
3	125.7, C	
4	177.9, C	
4a	118.7, C	
5	105.5, CH	7.60, s
6	149.7, C	
7	156.8, C	
8	101.2, CH	7.16, s
1'	125.7, C	
2', 6'	131.6, CH	7.51, d (8.8)
3', 5'	115.0, CH	7.01, d (8.8)
4'	161.3, C	7.02, d (8.8)
6-OCH$_3$	56.8, CH$_3$	3.96, s
7-OCH$_3$	57.2, CH$_3$	4.00, s

Table S3. NMR data of 5'-hydroxy-2',3',4,4''-tetramethoxy-p-terphenyl **(65)** in CD_3OD-d_4 (^{1}H 700 MHz, ^{13}C 176 MHz)

pos.	δ_C, type	δ_H,mult (*J* in Hz)
1	127.6, C	
2, 6	133.1, CH	7.35, d (8.8)
3, 5	114.3, CH	6.98, d (8.8)
4, 4''''	160.3, C	
1'	128.8, C	
2'	151.7, C	
3'	147.8, C	
4'	124.7, C	
5'	142.7, C	
6'	110.3, CH	6.73, s
1''	132.5, C	
2'', 6'''	131.5, CH	7.59, d (8.8)
3''', 5''	114.6, CH	6.99, d (8.8)
4-OCH_3, 4''-OCH_3	65.3, CH_3	3.86, s
2'-OCH_3	57.2, CH_3	3.68, s
3'-OCH_3	60.8, CH_3	3.35, s
